# The SOD1 Inhibitor, LCS-1, Oxidizes H2S to Reactive Sulfur Species, Directly and Indirectly, through Conversion of SOD1 to an Oxidase

**DOI:** 10.3390/antiox13080991

**Published:** 2024-08-15

**Authors:** Kenneth R. Olson, Tsuyoshi Takata, Kasey J. Clear, Yan Gao, Zhilin Ma, Ella Pfaff, Karthik Mouli, Thomas A. Kent, Prentiss Jones, Jon Fukuto, Gang Wu, Karl D. Straub

**Affiliations:** 1 Department of Physiology, Indiana University School of Medicine South Bend, South Bend, IN 46617, USA; tsuyoshi.takata.a5@tohoku.ac.jp (T.T.); yangao@iu.edu (Y.G.); zma3@nd.edu (Z.M.); epfaff@nd.edu (E.P.); 2 Department of Biological Sciences, University of Notre Dame, Notre Dame, IN 46556, USA; 3 Department of Chemistry and Biochemistry, Indiana University South Bend, South Bend, IN 46615, USA; kclear@iu.edu; 4 Institute of Biosciences and Technology, Texas A&M Health Sciences Center-Houston Campus, Houston, TX 77030, USA; kmouli@exchange.tamu.edu (K.M.); tkent@tamu.edu (T.A.K.); 5 Toxicology Department, Western Michigan University Homer Stryker M.D. School of Medicine, Kalamazoo, MI 49007, USA; prentiss.jones@wmed.edu; 6 Department of Chemistry, Sonoma State University, Rohnert Park, CA 94928, USA; fukuto@sonoma.edu; 7 Department of Internal Medicine, McGovern Medical School, University of Texas, Houston, TX 77030, USA; gang.wu@uth.tmc.edu; 8 Central Arkansas Veteran’s Healthcare System, Little Rock, AR 72205, USA; karl.straub@va.gov; 9 Departments of Medicine and Biochemistry, University of Arkansas for Medical Sciences, Little Rock, AR 72202, USA

**Keywords:** reactive sulfur species, reactive oxygen species, antioxidants, SOD1, SOD2

## Abstract

LCS-1, a putative selective inhibitor of SOD1, is a substituted pyridazinone with rudimentary similarity to quinones and naphthoquinones. As quinones catalytically oxidize H_2_S to biologically active reactive sulfur species (RSS), we hypothesized LCS-1 might have similar attributes. Here, we examine LCS-1 reactions with H_2_S and SOD1 using thiol-specific fluorophores, liquid chromatography–mass spectrometry, electron paramagnetic resonance (EPR), UV–vis spectrometry, and oxygen consumption. We show that LCS-1 catalytically oxidizes H_2_S in buffer solutions to form RSS, namely per- and polyhydrosulfides (H_2_S_n_, n = 2–6). These reactions consume oxygen and produce hydrogen peroxide, but they do not have an EPR signature, nor do they affect the UV–vis spectrum. Surprisingly, LCS-1 synergizes with SOD1, but not SOD2, to oxidize H_2_S to H_2_S_3-6_. LCS-1 forms monothiol adducts with H_2_S, glutathione (GSH), and cysteine (Cys), but not with oxidized glutathione or cystine; both thiol adducts inhibit LCS-1-SOD1 synergism. We propose that LCS-1 forms an adduct with SOD1 that disrupts the intramolecular Cys^57^-Cys^146^ disulfide bond and transforms SOD1 from a dismutase to an oxidase. This would increase cellular ROS and polysulfides, the latter potentially affecting cellular signaling and/or cytoprotection.

## 1. Introduction

The small-molecule lung cancer screen-1 (LCS-1) is a 4,5-dichloro-3(2*H*)-pyridazinone compound originally identified from a screen of 189,290 small molecules based on its ability to retard growth of select human lung adenocarcinoma cell lines with *EGFR* and *KRAS* mutations [1]. It was subsequently shown to be a potent and specific inhibitor of superoxide dismutase 1 (SOD1 [2]). Since then, targeting SOD1 with LCS-1 has proven to be an effective strategy against multiple myeloma cells [3], glioma tissue [4], colorectal cancer cells [5,6], and breast cancer cells [7]. Conversely, LCS-1 decreased the efficacy of the antioxidant flavonoid, dihydromyricetin, in reactive oxygen species (ROS)-sensitive hepatoblastoma cells [8].

The effects of LCS-1 are generally accepted to be mediated through an increase in ROS resulting from SOD1 inhibition. However, Steverding and Barcelos observed that LCS-1 was also effective against the protozoan parasite *Trypanosoma brucei*, even though *T. brucei* lacks SOD1 [9]. Although they did not pursue alternative mechanisms, their study suggests LCS-1 may have effects beyond SOD1 inhibition.

We have shown that SOD1 oxidizes hydrogen sulfide (H_2_S) to hydrogen per- and polysulfides (H_2_S_n_, where n = 2–5), collectively referred to as reactive sulfur species (RSS), and it has recently been proposed that SOD1 is a major pathway for H_2_S metabolism in cells [10]. We have also shown that it is difficult to analytically distinguish between the most biologically relevant ROS, i.e., hydrogen peroxide (H_2_O_2_) and superoxide (O_2_^−^), and the RSS, as well as hydrogen persulfide (H_2_S_2_) and supersulfide S_2_^−^ [11]. Furthermore, ROS and RSS signal via common pathways; H_2_O_2_ sulfenylates and H_2_S_2_ persulfidates redox-sensitive cysteines on many regulatory proteins; and ROS and RSS initiate identical responses. However, H_2_S_2_ tends to be more efficacious than H_2_O_2_ [12,13].

**Figure 1:**
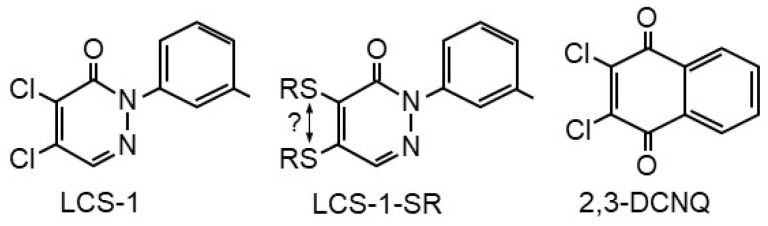
Structure of LCS-1, LCS-1-thiol adducts (R = H, GSH, or cysteine), and 2,3-dichloro-1,4-naphthoquinone.

Given the fact that SOD1 oxidizes H_2_S to H_2_S_2_ and that LCS-1 is a potent SOD1 inhibitor, we initially hypothesized that LCS-1 would increase cellular H_2_S and decrease H_2_S_2_. However, our initial studies in cell-free systems suggested that LCS-1 did not inhibit SOD1 oxidation of H_2_S, but it augmented it. Furthermore, LCS-1 appeared to directly oxidize H_2_S in the absence of SOD1. Although LCS-1 is not a quinone, it has some structural resemblance to 2,3-dichloro-1,4-naphthoquinone (DCNQ; Figure 1), and we have shown that DCNQ and a variety of other naphthoquinones (NQs) catalytically oxidize H_2_S to H_2_S_n_ [14,15]. However, to the best of our knowledge, no redox cycling activity has been reported for LCS-1 or other molecules of the 4,5-dichloro-3(2*H*)-pyridazinone class of compounds. In the present work, we examine the catalytic properties of H_2_S metabolism by LCS-1 guided by our experience with H_2_S and NQs.

## 2. Methods

### 2.1. H_2_S and Polysulfide Measurements

AzMC (7-azido-4-methylcoumarin, 365/450 nm Ex/Em) and SSP4 (3’,6’-Di(O-thiosalicyl)fluorescein, 482/515 nm Ex/Em) fluorophores were used to detect H_2_S and per/polysulfides (H_2_S_n_, n = 2–7). These fluorophores are specific for H_2_S and H_2_S_n_, respectively, and insensitive to other sulfur compounds, ROS, and reactive nitrogen species [16,17,18]. Fluorophores and reactants were mixed in 96-well plates, and fluorescence was measured with a SpectraMax M5e plate reader (Molecular Devices, Sunnyvale, CA, USA). Plates were covered with tape to minimize H_2_S volatilization. As both AzMC and SSP4 are irreversible, they provide a cumulative record of H_2_S and polysulfide production.

### 2.2. Liquid Chromatography Mass Spectrometry (LC-MS/MS)

LC-MS/MS analysis was conducted using a Waters Micromass Quattro Premier Triple Quadropole Mass Spectrometer coupled to the Waters Alliance 2795 LC system. Chromatographic separation was accomplished using a YMC-Triart C18 column with dimensions of 50 × 2.1 mm inner diameter with a mobile phase consisting of A (0.1% formic acid) and B (0.1% formic acid in methanol) with a flow rate of 0.2 mL/min at 40 °C. Ten μL were injected with a linear gradient (5–90% B, 0–4 min, and 90% B, 4–7 min). The mass spectrometer was operated using electrospray ionization in positive ion mode with the capillary voltage set to 3500 V and drying gas set to 10.0 L/min at 350 °C. Moreover, β-(4-hydroxyphenyl)ethyl iodoacetamide (HPE-IAM, [19]) polysulfide adducts were detected as the [M + H]^+^ ion using their exact masses of ± 0.002 m/z: S_1_ (389.153), S_2_ (421.125), S_3_ (453.097), S_4_ (485.069), S_5_ (517.041), S_6_ (549.013), S_7_ (580.985), HSO_3_ (260.059), HS_2_O_3_ (292.031), HS_4_O (323.985), HS_5_O (355.957).

In a typical experiment, a solution containing 100 μM Na_2_S, 1 μM SOD, or 10 μM LCS-1 was incubated at room temperature for 2 h. Following this incubation period, HPE-IAM was introduced to achieve a final concentration of 5 mM. The mixture underwent an additional incubation at 37° for 20 min. The resulting reaction mixtures were analyzed using LC-MS/MS with selected ion recording (SIR).

Polysulfides and sulfoxides can be derived directly from LCS-1-SOD1 oxidation of H_2_S or indirectly by SOD1 dismutation of superoxide to H_2_O_2_, which then oxidizes H_2_S. To examine these possibilities, 100 μM of H_2_S was incubated for 2h with 1 μM SOD1 and 10 μM LCS-1 without or with 1 μM catalase and subjected to LC-MS/MS analysis.

For cell experiments, HEK293T cells (kindly provided by Dr. X. Lu, University of Notre Dame, purchased from American Type Culture Collection, Manassas, VA, USA) were cultured for 48 h in 21% or 5% O_2_ in Dubelco’s medium (Life Technologies Corp. Grand Island, NY, USA) without or with 0.3 μM and 1 μM LCS-1. The medium was removed, the cells were washed 2 times with PBS, and 5 mM HPE-IAM/70% methanol/30 mM acetate buffer (pH 6.5) (0.4 mL/dish) was added. The cells were collected, sonicated (power output 4, 10 s), and incubated at 37° for 20 min. They were then centrifuged (10,000× *g*, 10 min), and the supernatants were diluted by 0.1% FA to 1/2 and analyzed by LC-MS/MS.

### 2.3. Colloidal Sulfur (S_8_)

Progressive catenation of polysulfides to cyclic S_8_ forms an insoluble, turbid colloid over 10–30 min that can be detected by comparing optical density at 263 nm (OD_263_), where light is also absorbed by soluble sulfur compounds to OD at 650 nm (OD_650_), which is only affected by turbidity. To determine if a detectable level of S_8_ was formed in our experiments, 300 μM H_2_S was incubated with 10 μM LCS-1 without or with 1 μM SOD in air-saturated buffer, pH 7.4, 22 °C, and OD monitored for 30 min.

### 2.4. Oxygen Consumption

Oxygen tension was monitored in a stirred 1 mL water-jacketed chamber at room temperature with a FireStingO_2_ oxygen-sensing system (Pyroscience Sensor Technology, Aachen, Germany) and a non-oxygen-consuming, 3 mm diameter OXROB10 fiberoptic probe. The probe was calibrated with room air (21% O_2_) or nitrogen gas (0% O_2_). Compounds of interest were added to PBS buffer containing 50 μM of desferrioxamine at timed intervals, and percent oxygen (100% equals room air) was measured every 0.1–0.3 s for 60 min. Oxygen concentration (in μM) was estimated by multiplying the percent oxygen by the solubility coefficient for oxygen in 300 mOsm saline at 20 °C (2.65 μmol⋅L^−1^⋅% O_2_^−1^, i.e., for air-saturated buffer, 2.65 × 100 = 265 μmol oxygen). Oxygen consumption was calculated from the difference between the oxygen concentration immediately after adding the compounds of interest and the oxygen concentration at the response nadir.

### 2.5. H_2_O_2_ Detection with Amplex Red

LCS-1, 50 mM in DMSO, was diluted into PBS, pH 7.4 to 25 uM. Amplex Red and horseradish peroxidase were then added with final concentrations of 9.4 uM and 0.02 μM, respectively. Then, one equivalent (25 μM) of H_2_S as Na_2_S was added, and the oxidation of Amplex Red was followed by the change in A570. Catalase (0.028 μM) or SOD (0.1 μM) was included in parallel experiments.

### 2.6. SOD1 Assay

Two methods were used to measure the effects of LCS-1 on SOD1. In the first, SOD1 activity was measured using the method of Ewing and Janero [20], where superoxide is produced by the reaction of phenazine methosulfate (PMS) with NADH. This method is independent of other enzymes that could potentially react with H_2_S or polysulfides. Moreover, 200 μL of reaction buffer (0.1 mM EDTA, 62 μM nitro blue tetrazolium, and 100 μM NADH in 50 mM phosphate buffer (PBS)) were placed in 96-well plates without or with various concentrations of SOD. Furthermore, 25 μL of 150 μM PMS with 0.1 mM EDTA in 50 mM PBS were added, and absorbance at 560 nm was measured after 5 min on the plate reader. The effect of LCS-1 or the copper chelator ATN-244 (2-hydroxy-N,N,N-trimethylethanaminium tetrathiomolybdate) [2] was examined by incubating 1 μM SOD in reaction buffer with various concentrations of LCS-1 and/or ATN-244 for 30 min prior to addition of PMS.

For comparison, a method similar to that used by Somwar et al. [2] to measure SOD was also examined. This method uses the tetrazolium dye, WST1, for the detection of superoxide radicals generated from the reaction catalyzed by xanthine in the presence of xanthine, hypoxanthine, and diatomic oxygen. SOD1 inhibits the reaction of the tetrazolium indicator with superoxide radicals and attenuates the increase in optical absorbance. Briefly, bovine erythrocyte-derived SOD1 at a final well concentration of 0.2 U/mL was incubated with varying concentrations of LCS-1 in DMSO for 20 min at 25 °C while shaken at 500 rpm. The DMSO concentration of each well was kept constant at 0.417% by volume. Following incubation, 20 µL of the SOD1-LCS-1 solution were added to 200 µL of assay buffer solution containing WST1 in a 96-well plate and shaken for 10 min at room temperature to ensure adequate mixing. Twenty µL of assay initiation buffer containing xanthine oxidase was added to each well, and the plate was incubated at 37 °C for 10 min. Optical absorbance was measured at 450 nm using a microplate reader (CLARIOstar Plus, BMG Labtech (US), Cary, NC, USA), and absorbance values were converted to SOD1 activity units per assay manufacturer’s instructions.

### 2.7. Electron Paramagnetic Resonance (EPR) Spectrometry

X-band EPR spectra were recorded at 295 K on a Bruker EMX spectrometer (Billerica, MA, USA). The instrument parameters were frequency, 9.30 GHz; MW power, 4 mW; range, 20 G; modulation frequency, 100 kHz; modulation amplitude, 0.2 G; and time constant, 0.17 s. The simulation was conducted using Simfonia (Bruker).

EPR samples were prepared by diluting 4 mM LCS-1 stock in DMSO into PBS buffer (pH 7.4, final LCS-1 concentration, 1 mM). Equimolar concentrations of H_2_S, ferricyanide NaBH_4_, or dithionite crystals were added, and the reaction solutions were then transferred into capillary tubes for EPR measurements. H_2_S was prepared by dissolving Na_2_S in N_2_ sparged PBS.

### 2.8. Absorbance Spectra

Absorbance spectra were measured with an Agilent HP 8453 spectrometer (Agilent Technologies, Santa Clara, CA, USA). In a typical experiment, the reagents were dissolved in a 50 mM DMSO stock and spectra obtained at intervals over at least 25 min. The DMSO spectrum was subtracted to minimize interference. The absorption spectra of the LCS-1 ferricyanide experiment were conducted in tandem cells; initially, 100 μM LCS-1 was placed in one cell, and the spectrum was obtained. Ferricyanide (250 μM) was placed in the other cell and a second spectrum obtained, then the samples were mixed, and spectra obtained immediately and 10 min after mixing.

### 2.9. Preparation of Thiol Adducts

GSH and Cys adduct formation was examined by incubating 10 μM LCS-1 and 0.1 μM SOD1 with either 10 μM or 50 μM glutathione (GSH) or cysteine (Cys) for 30 min at room temperature. They were then aliquoted into 96-well plates, and SSP4 (10 μM) and 300 μM H_2_S were added and counted on the plate reader. H_2_S adducts were prepared by incubating 10 μM LCS-1 with either 10 μM or 50 μM H_2_S for 30 min in covered containers, after which the cover was removed and left uncovered for 2 h to allow the free H_2_S to dissipate through volatilization. The LCS-1-H_2_S adducts were then added to 96-well plates, followed by SOD1 and 300 μM H_2_S, and counted.

### 2.10. Speciation of Inorganic RSS

The percent ionization of inorganic RSS (H_2_S_n_, HS_n_^−^, and S_n_^2−^) was calculated at pH increments from 3 to 10 by solving simultaneous Henderson–Hasselbach equations for the respective pKa_1_ and pKa_2_ as described in Supplemental Information. The percent of each species was then plotted as a function of pH using SigmaPlot (Systat Software, San Jose, CA, USA).

### 2.11. Chemicals

SSP4 and the SOD assay kit were purchased from Dojindo Molecular Technologies Inc. (Rockville, MD, USA). LCS-1 and all other chemicals were purchased from Sigma-Aldrich (St. Louis, MO, USA) or ThermoFisher Scientific (Grand Island, NY, USA). Unless otherwise specified, ‘H_2_S’ is used throughout to denote the total sulfide (sum of dissolved H_2_S, hydrosulfide anion, HS^−^, and dianion S^2−^). Phosphate-buffered saline (PBS; in mM): 137 NaCl, 2.7 KCl, 8 Na_2_HPO_4_, 2 NaH_2_PO_4_ was adjusted to pH 7.4. H_2_S solutions were prepared in PBS sparged for at least 20 min with nitrogen gas.

### 2.12. Statistical Analysis

Data were analyzed and graphed using QuattroPro (Corel Corporation, Ottawa, ON, Canada) and SigmaPlot 13.0 (Systat Software, Inc., San Jose, CA, USA). Statistical significance was determined with the Student’s t-test or one-way ANOVA and the Holm–Sidak test for multiple comparisons as appropriate using SigmaStat (Systat Software, San Jose, CA, USA). Results are given as mean +/− SE; significance was assumed when *p* < 0.05.

## 3. Results

### 3.1. LCS-1 Synergizes with SOD1 but Not SOD2 to Oxidize H_2_S to Polysulfides

**Figure 2:**
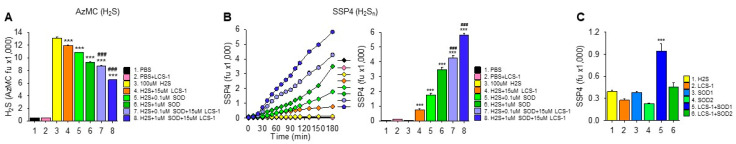
LCS-1 and SOD1, but not SOD2, synergistically consume H_2_S to produce polysulfides. (**A**) Both LCS-1 and SOD1 consume H_2_S (AzMC fluorescence), and when LCS-1 and SOD1 are combined, H_2_S consumption is further increased. H_2_S consumption was measured by incubating 100 μM H_2_S with 15 μM LCS-1 or with either 0.1 μM or 1 μM bovine SOD1 with or without 15 μM LCS-1 for 2 h; 25 μM AzMC was then added, and fluorescence was measured. Mean +SE, n = 4; ***, *p* < 0.001 compared to H_2_S; ###, *p* < 0.001 compared to respective SOD1. (**B**) Both LCS-1 and SOD1 produce polysulfides (SSP4 fluorescence), and when combined, polysulfide production is further increased. Polysulfide production was measured by incubating 25 μM SSP4 with 300 μM H_2_S and with 15 μM LCS-1 or with H_2_S and 0.1 μM or 1 μM SOD1 alone, or in combination with 15 μM LCS-1. SSP4 fluorescence was monitored at 10 min intervals for 120 min (left panel); right panel summarizes results at 180 min; mean +SE, n = 4; ***, *p* < 0.001 compared to H_2_S; ###, *p* < 0.001 compared to respective SOD1. (**C**) SOD2 does not synergize with 10 μM LCS-1 to oxidize 300 μM H_2_S to polysulfides (SSP4 fluorescence). SOD1 and SOD2 0.1 μM, mean + SE, *** *p* < 0.001 vs. H_2_S.

Both SOD1 and SOD2 catalytically oxidize H_2_S to polysulfides [21]. To determine if LCS-1 inhibited this reaction, 100 μM H_2_S was incubated with either 0.1 μM or 1 μM bovine SOD1 with or without 15 μM LCS-1 for 2 h, then 25 μM AzMC was added to determine H_2_S consumption. It was hypothesized that LCS-1 would decrease H_2_S catabolism, but surprisingly, LCS-1 alone slightly increased H_2_S consumption. This effect was more than additive, with SOD1 augmenting H_2_S consumption by around 50% (Figure 2A). To determine if the H_2_S-LCS-1 reaction produced polysulfides, 300 μM H_2_S was incubated with SOD1 and LCS-1, and polysulfide production was measured with SSP4. Both LCS-1 and SOD1 oxidized H_2_S to polysulfides, but when combined, they appeared to augment polysulfide production (Figure 2B). Conversely, LCS-1 did not appear to synergize with SOD2 to oxidize H_2_S to polysulfides (Figure 2C).

**Figure 3:**
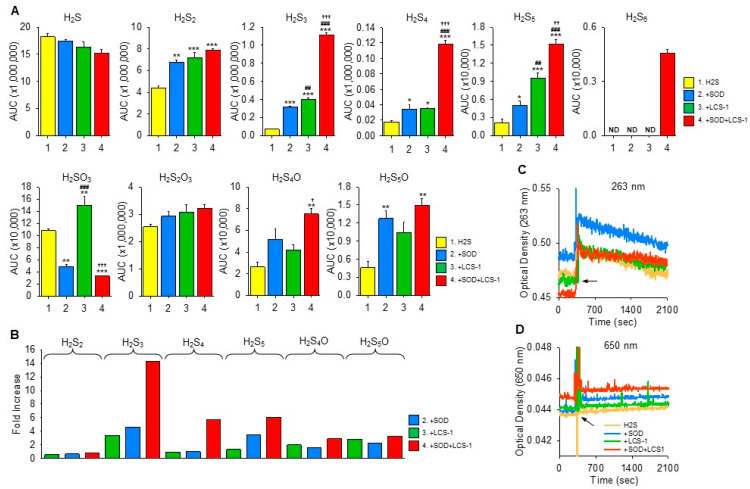
(**A**) LC-MS/MS analysis (area under the curve, AUC) of reactive sulfur species in 100 μM H_2_S and after 20 min incubation of H_2_S with 1 μM SOD1, 10 μM LCS-1, and SOD1 with LCS-1. SOD1, LCS-1, and SOD1 with LCS-1 progressively decreased H_2_S; only SOD1 increased H_2_S_2_, whereas LCS-1 alone or in combination with SOD1 increased H_2_S_3_ and H_2_S_4_. Conversely, SOD1 alone and in combination with LCS-1 decreased sulfite (H_2_SO_3_), whereas LCS-1 had no effect. Thiosulfate (H_2_S_2_O_3_) was unaffected by any treatment. Sulfenic acids H_2_S_4_O and H_2_S_5_O were increased by SOD plus LCS-1, and SOD increased H_2_S_5._ Mean +SE, n = 3 replicates; *, *p* < 0.05; **, *p* < 0.01; ***, *p* < 0.001 vs. H_2_S; ##, *p* < 0.01; ###, *p* < 0.001 SOD1 vs. LCS-1 or SOD1 + LCS-1; †, *p* < 0.05; ††, *p* < 0.01; †††, *p* < 0.001 LCS-1 vs. SOD1 + LCS-1. (**B**) Fold increase in SOD1, LCS-1, and SOD1 plus LCS-1 compared to H_2_S control (e.g., AUC_SOD1_/AUC_H2S_). (**C**,**D**) Optical density (OD) at OD_263_ and OD_650_ following addition of 10 μM LCS-1, without or with 1 μM SOD and 300 μM H_2_S at approximately 300 s (arrows). All combinations produced a rapid increase in OD_263_ followed by a slow, linear decrease, there was no notable change in OD_650_ suggestive of little or no S_8_ production.

The products of H_2_S oxidation by SOD1, LCS-1, and SOD1 with LCS-1 in buffer were then examined by LC-MS/MS analysis (Figure 3A). SOD1, LCS-1, and SOD1 with LCS-1 progressively but not significantly decreased H_2_S. SOD1, LCS-1, and SOD1 plus LCS-1 increased H_2_S_2_ compared to H_2_S alone, but there was no difference between the three. SOD1, LCS-1, and SOD1 plus LCS-1 increased H_2_S_3_, H_2_S_4_, and H_2_S_5_, and the combination of SOD1 and LCS-1 increased H_2_S_3_ and H_2_S_4_ more than the sum of SOD1 and LCS-1. Only the combination of SOD1 and LCS-1 produced a detectable amount of H_2_S_6_. SOD1 and SOD1 plus LCS-1 decreased sulfite (H_2_SO_3_) compared to control, whereas LCS-1 increased it. Thiosulfate (H_2_S_2_O_3_) was unaffected by any treatment. A small amount of S_4_ and S_5_ sulfenic acids were also detected, and both were increased by the combination of SOD1 and LCS-1. H_2_S_5_O was also increased by SOD1 alone. The fold increase in SOD1, LCS-1, and SOD1 plus LCS-1 compared to H_2_S control (AUC_SOD1_/AUC_H2S_ from Figure 3A) is shown in Figure 3B. The combination of SOD1 and LCS-1 had the greatest effect on H_2_S_3_, H_2_S_4_, and H_2_S_5_ and on both sulfenic acids (note: the fold increase for H_2_S_6_ would be even greater but was not calculated as H_2_S_6_ was not detected when H_2_S was incubated with either SOD1 or LCS-1 alone).

These results provide additional evidence that LCS-1 oxidizes H_2_S to select polysulfides. They also confirm earlier observations that SOD oxidizes H_2_S to polysulfides. Most surprising, however, was the observation that the amount of polysulfides produced by combining SOD1 and LCS-1 was more than additive. This would not be expected if LCS-1 was inhibiting the H_2_S oxidative capacity of SOD1. It is also interesting that some residual H_2_SO_3_ in H_2_S was decreased by SOD1 and SOD1 + LCS-1 but increased by LCS-1. This suggests that some of the enzymatic attributes of SOD1 are not inhibited and distinct from LCS-1-SOD1 interactions.

### 3.2. Oxidation of H_2_S by LCS-1 Does Not Produce Colloidal Sulfur (S_8_)

The addition of H_2_S to LCS-1, without or with SOD, produced a rapid increase in OD_263_ followed by a slow, linear decrease over the ensuing 30 min (Figure 3C). There was no notable change in OD_650_ in any of the reactions (Figure 3D). This suggests that either there is a relatively rapid production of some sulfur compound or that the initial increase in absorbance is due to H_2_S and polysulfide contaminants, but little if any S_8_ colloid appeared to be formed.

### 3.3. Effects of SOD1 and LCS-1 Concentrations on Synergistic Oxidation of H_2_S

**Figure 4:**
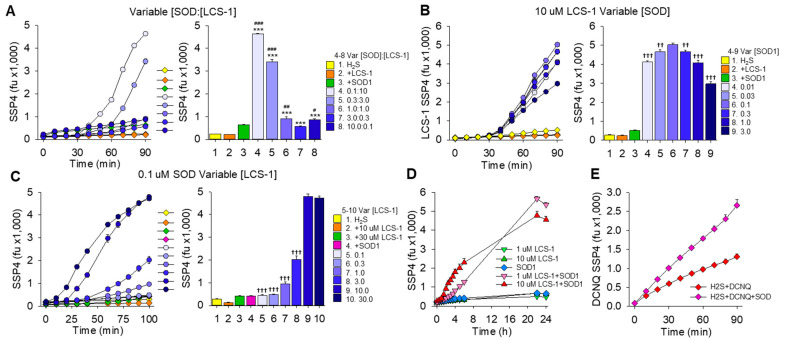
Effects of LCS-1 and SOD1 concentration on synergistic oxidation of H_2_S to polysulfides (SSP4 fluorescence). (**A**) Incubation of 300 μM H_2_S with 10 μM LCS-1 or μM SOD1 or with various concentration ratios of SOD1 to LCS-1 ([SOD1]:[LCS-1]). LCS-1 alone did not affect polysulfides compared to H_2_S alone, whereas SOD1 increased it (*p* < 0.001). All combinations of SOD1 and LCS-1 SOD1 increased polysulfides compared to H_2_S with 10 μM LCS-1 (***, *p* < 0.001), and all but 3.0 μM SOD:3.0 μM LCS-1 increased polysulfides compared to 10 μM SOD1 (#, *p* < 0.05; ##, *p* < 0.01; ###, *p* < 0.001). The 0.1:1.0 SOD1:LCS-1 combination produced more (*p* < 0.001) polysulfides than any other combination of SOD1 and LCS-1. (**B**) Effects of 10 μM LCS-1 and variable SOD concentrations on polysulfides produced by 300 μM H_2_S compared to H_2_S and 10 μM LCS-1 or 10 μM SOD1. All combinations of SOD and LCS-1 produced more polysulfides than with either LCS-1 or SOD1 alone (*p* < 0.001). More polysulfides were produced by 0.1 μM SOD1 and 10 μM LCS-1 than by any other combination of SOD1 and LCS-1 (††, *p* < 0.01; †††, *p <* 0.001). (**C**) Effects of 10 μM SOD1 and variable LCS-1 concentrations on polysulfides produced by 300 μM H_2_S compared to H_2_S and either 10 μM SOD1 or 10 μM or 30 μM LCS-1 alone. Combinations of SOD and LCS-1 produced more polysulfides than with either LCS-1 or with SOD1 alone (*p* < 0.001). More polysulfides were produced by 0.1 μM SOD1 and either 10 μM or 30 μM LCS-1 than by any other combination of SOD1 and LCS-1; there was no difference between 0.1 μM SOD and either 10 μM or 30 μM LCS-1 (†††, *p* < 0.001; 0.1 μM SOD and 10 μM LCS-1 vs. other SOD-LCS-1 combinations). (**D**) Polysulfide production after incubation of 0.1 μM SOD1 and 300 μM H_2_S with either 1 μM or 10 μM LCS-1 for 24 h. The initial delay in polysulfide production was longer with 1 μM LCS-1 up to 6 h, but by 22 h there was no difference between 1 μM and 10 μM LCS-1; mean +SE, n = 4 replicates. (**E**) Polysulfide production by addition of 300 μM H_2_S to 3 μM 2,3-dichloro-1,4-naphthoquinone (DCNQ) without or with 0.1 μM SOD1. SOD increased polysulfide production, but there was no obvious lag period in either reaction. All samples are mean +SE, n = 4 wells; right panels summarize values at 90 min.

Synergism between SOD1 and LCS-1 was further examined by incubating H_2_S with various concentrations of LCS-1 and SOD1 (Figure 4). As shown in Figure 4A, varying the ratio of SOD1 to LCS-1 produced a large increase in polysulfides at the lowest (0.1 μM) SOD concentration and highest (10 μM) LCS-1 concentration (0.1:10.0 [SOD1]:[LCS-1]). Polysulfide production then decreased as the [SOD]:[LCS-1] concentration ratio increased until at and above 1:1 [SOD1]:[LCS-1], when polysulfide levels were only slightly above those produced by H_2_S and 0.1 μM SOD1. Increasing SOD1 with LCS-1 at 10 μM (Figure 4B) produced a near-maximal response at 0.01 μM SOD1, with further, albeit slight, increases at 0.03 μM and 0.1 μM. Polysulfide production then decreased as the SOD1 concentration increased. Increasing LCS-1 with SOD1 at 0.1 μM (Figure 4C) produced a concentration-dependent increase in polysulfides up to 10 μM LCS-1; there was no difference between 10 μM and 30 μM LCS-1. These results show that there is a concentration-dependent relationship in SOD1-LCS-1 synergism that is sensitive to both SOD1 and LCS-1 concentrations. This synergism requires very little SOD1 on a molar basis, but at least a ten-fold excess of LCS-1 appeared to be necessary to obtain the maximum H_2_S catalytic activity. To determine if the effect of LCS-1 concentration was due to a molar requirement for LCS-1 or that there was just more delay with the lower concentration, the oxidation of H_2_S and SOD1 with either 1 μM or 10 μM LCS-1 was monitored for 24 h (Figure 4D). The delay with 1 μM LCS-1 was clearly longer than that with 10 μM LCS-1 over the initial 6 h, but by 22 h there was no difference in polysulfide production between 1 μM or 10 μM LCS-1. This suggests that the delay is the major factor in the apparent concentration-dependent sensitivity to LCS-1. By comparison, there was no noticeable lag period when H_2_S was added to 2,3-dichloro-1,4-naphthoquinone (DCNQ) either without or with SOD1 (Figure 4E). This further differentiates the effects of LCS-1 from those of DCNQ.

### 3.4. H_2_S Oxidation by SOD1 and LCS-1 Is Oxygen-Dependent and Produces Hydrogen Peroxide

**Figure 5:**
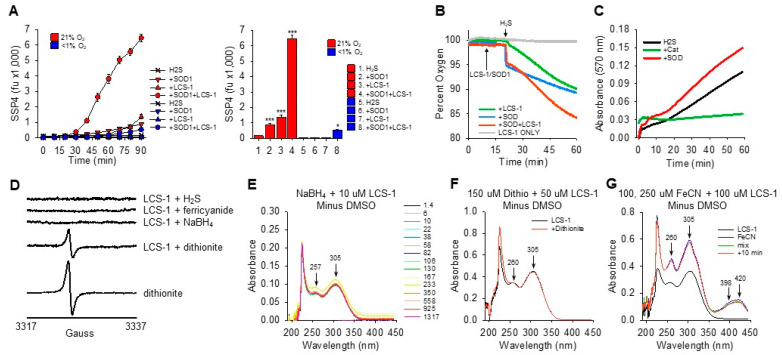
Characteristics of H_2_S oxidation by SOD1 and LCS-1. (**A**) Oxygen dependency of polysulfide production (SSP4 fluorescence) on oxidation of 300 μM H_2_S by 0.1 μM SOD1 and 10 μM LCS-1 alone or in combination in 21% oxygen (room air) or <1% oxygen. No polysulfides were produced in <1% oxygen by H_2_S with SOD1 or LCS-1, and polysulfides were decreased more than ten-fold by SOD1 and LCS-1 together. Right panel shows 90 min mean +SE, n = 4 wells; * *p* < 0.05, ***, *p* < 0.001 compared to respective control. (**B**) Oxygen consumption following addition of 300 μM H_2_S to 1 μM SOD1 and 10 μM LCS-1 alone and in combination. Adding H_2_S to SOD1 produced a rapid decrease in O_2_, followed by a steady decline. H_2_S added to LCS-1 produced a slight, rapid decrease in O_2_, followed by what appeared to be a two-phase decrease that started slowly and then increased after 15 min. H_2_S added to LCS-1 with SOD1 produced a rapid drop in oxygen, followed by a two-phase decrease that consumed more oxygen than the sum of the individual H_2_S-LCS-1 and H_2_S-SOD1. No oxygen was consumed by LCS-1 alone. (**C**) Addition of 25 μM H_2_S to 25 μM LCS-1 continually produces H_2_O_2_. H_2_O_2_ production was inhibited by 0.028 μM bovine catalase but augmented by 0.1 μM SOD (Amplex Red assay; traces show absorbance at 570 nm). (**D**) EPR spectra of 4 mM LCS-1 added to 4 mM H_2_S, 4 mM ferricyanide, several crystals of NaBH_4_, 4 mM dithionite, or 4 mM dithionite alone. There was no evidence of LCS-1 radicals. (**E**–**G**) Time-resolved absorbance spectra of LCS-1 reactions with reductants and oxidants; the spectrum of DMSO in solvent was subtracted for clarity. (**E**) Spectrum of 10 μM LCS-1 following addition of several crystals of sodium borohydride (NaBH_4_) at 22 s. (**F**) Spectrum before and after addition of 150 μM dithionite (S_2_O_4_^2−^) to 50 μM LCS-1. (**G**) Spectra in tandem cuvettes, 100 μM LCS-1 only in cuvette #1 (black line), 150 μM ferricyanide (FeCN) only in cuvette #2 (blue line), immediately or 10 min after mixing cuvettes 1 and 2 (green and red lines, respectively). There was no effect of any compound on the LCS-1 spectrum.

Oxygen dependency of H_2_S oxidation to polysulfides (SSP4 fluorescence) by SOD1 and LCS-1 was examined by sparging the reagents with nitrogen for 20 min prior to mixing and covering the 96-well plates with tape during counting. This reduced oxygen tension to <1%, prevented polysulfide formation by both SOD1 and LCS-1, and almost eliminated polysulfide production by SOD1 combined with LCS-1 (Figure 5A).

To confirm the oxygen dependency of these reactions, oxygen consumption was monitored following the addition of H_2_S to LCS-1, SOD1, and the combination of LCS-1 and SOD1. As shown in Figure 5B, the addition of H_2_S produced a slight, rapid decrease in oxygen tension followed by a steady decline. Adding H_2_S to SOD1 produced a rapid 5% decrease in oxygen tension, equivalent to a decrease of 13 μM, followed by a steady decline that was similar to that of H_2_S alone. H_2_S added to LCS-1 produced a slight (<1%) rapid decrease in oxygen, followed by what appeared to be a two-phase decrease that started slowly and then increased after 15 min. The rate of oxygen decrease after 15 min was approximately 0.23%, or 0.62 μmoles/min, and it exceeded the rate of H_2_S alone or H_2_S with SOD1. Adding H_2_S to SOD1 combined with LCS-1 produced a curve that exhibited characteristics of both SOD1 and LCS-1, although the net decrease in oxygen (24%, 63 μmoles) was greater than the sum of each of the two compounds (10% each, 53 μmoles total). The increase in oxygen consumption approximately 15 min after addition of H_2_S to SOD1 combined with LCS-1 is consistent with the delayed increase in polysulfide production observed in Figure 4.

Using the Amplex Red assay to detect H_2_O_2_, it was evident that addition of H_2_S to LCS-1 produced a progressive increase in absorbance that was inhibited by catalase, confirming that H_2_O_2_ was produced by H_2_S and LCS-1 (Figure 5C). The rate of H_2_O_2_ production from the H_2_S-LCS-1 reaction was further increased by SOD1, and in both the H_2_S-LCS-1 and H_2_S-LCS-1-SOD1 reactions, there was a noticeable increase in the rate of H_2_O_2_ production after approximately 15 min. This supports our observations of a synergistic interaction between LCS-1 and SOD1 that has an initial slow and a delayed, more rapid second phase.

### 3.5. SOD1-LCS-1 Oxidation of H_2_S Does Not Appear to Involve Redox Cycling of LCS-1

LCS-1-catalyzed oxidation of H_2_S and reduction of oxygen to hydrogen peroxide is similar to previous observations of redox cycling in H_2_S/oxygen reactions with naphthoquinones. Naphthoquinones undergo two consecutive single-electron reduction/oxidation reactions with H_2_S and molecular oxygen, respectively. This produces a characteristic semiquinone radical, detectable by EPR, and it changes the UV–vis spectrum characteristics from that of an oxidized to a fully reduced NQ [14,22,23]. These methods were employed to determine if similar processes were involved in H_2_S/oxygen reactions catalyzed by LCS-1.

Addition of 4 mM H_2_S to 4 mM LCS-1 did not produce any detectable EPR signal, nor did addition of 4 mM ferricyanide, NaBH_4_ crystals, or 4 mM dithionite (Figure 5D). The EPR signature in the LCS-1-dithionite reaction comes from the decomposition of dithionite to the SO_2•_ radical, as shown in the bottom trace. These results suggest that either a radical species is not formed in LCS-1 H_2_S reactions or that it is too short-lived to be detected.

Similarly, no obvious changes were observed in UV–vis absorption spectra of reactions between 4 mM LCS-1 and 4 mM sodium borohydride (NaBH_4_), dithionite (S_2_O_4_^2−^) crystals, and 4 mM ferric cyanide (Fe(CN)_6_^3−^; Figure 5E–G). Absorption spectra of LCS-1 and reactions with H_2_S and Cys are shown in Supplemental Figure S1. H_2_S had two peaks at 209 nm and 229 nm. LCS-1 had two sharp peaks at 207 nm and 221 nm and two broad peaks at 257 nm and 306 nm. However, the sharp peaks were dominated by DMSO, and these disappeared when the DMSO spectrum was subtracted. The addition of 50 μM or 250 μM H_2_S to 50 μM LCS-1 produced a slight increase in the 221 nm peak, slightly red-shifted the downslope of the 221 nm peak and decreased the nadir at 245 nm. Higher concentrations of all reactants produced similar responses. The addition of SOD to LCS-1 and H_2_S produced a broad 305–322 nm peak that was assumed to be due to polysulfides. Cysteine (50 μM) did not affect the LCS-1 absorption spectrum. Collectively, these results suggest that, unlike NQs, there are no obvious redox-dependent changes in the LCS-1 spectrum that could be resolved within the time frame of our experiments.

### 3.6. Catalase Only Slightly Inhibits LCS-1-SOD1 Oxidation of H_2_S

The addition of catalase to the H_2_S-LCS-1-SOD1 reaction did not produce a consistent or substantial effect on the reaction products as measured by LC-MS/MS. Catalase slightly decreased H_2_S_2-4_ and H_2_SO_3_ and increased H_2_S_2_O_3_, whereas H_2_S, H_2_S_5,6_ and HS_4,5_OH were unaffected (Supplemental Figure S2). These results suggest that polysulfides and sulfoxides are primarily derived from the LCS-1-SOD1 oxidation of H_2_S and are not secondarily produced through H_2_O_2_ oxidation.

### 3.7. LCS-1 Forms Monothiol Adducts with SOD1 That Affect H_2_S Oxidation

GSH and Cys form adducts with NQs through Michael addition, and these adducts may affect NQ oxidation of H_2_S [14]. NQ-S adducts include mono- and di-thiol NQs and di-NQs connected to each other by NQ-Sn-NQ bridges (where S ≥ 1). However, LC-MS/MS examination showed that only H_2_S, glutathione, and cysteine monothiol S-adducts were formed with LCS-1, and this was through the replacement of one of the chloride atoms (Figure 1). While it was not possible to identify which chloride was involved, it has been reported that 4,5-dichloro-3(2*H*)-pyridazinones tend to react with sulfur nucleophiles at the 4-position (β to the carbonyl) under aqueous reaction conditions [24].

The effects of GSH and Cys adducts on H_2_S oxidation by LCS-1 and SOD1 were initially examined by incubating either thiol with LCS-1 and SOD1 for 30 min before adding SSP4 and H_2_S (Supplemental Figure S3A,B). Equimolar (10 μM) concentrations of LCS-1 and GSH increased polysulfide production, whereas 50 μM GSH and both 10 μM and 50 μM Cys decreased it. Neither GSH nor Cys affected polysulfide production when H_2_S was incubated with LCS-1 or SOD1 separately.

It is difficult to assess the effects of LCS-1-HS adducts on interactions with SOD1 because LCS-1 not only forms adducts with H_2_S but it also oxidizes H_2_S. Two sets of experiments were designed to address this issue. In the first set of experiments, 10 μM LCS-1 or 10 μM LCS-1 plus 0.1 μM SOD1 were pre-incubated for 10 min with either 10 μM or 50 μM H_2_S, then 300 μM H_2_S and SSP4 were added and counted for 140 min. These were compared to LCS-1 plus SOD pre-incubated without H_2_S for 0 or 10 min (Supplemental Figure S4A). Preincubation of LCS-1 with either 10 μM or 50 μM prevented polysulfide (SSP4 fluorescence) production. Simultaneous addition of LCS-1, SOD1, and 300 μM H_2_S produced a large increase in polysulfide production that was slightly but significantly greater than polysulfide production when LCS-1 and SOD1 were preincubated for 10 min before adding 300 μM H_2_S. Preincubation of LCS-1 or LCS-1 plus SOD1 with either 10 μM or 50 μM H_2_S consistently decreased polysulfide production from 75% to 40%, although 50 μM H_2_S was somewhat less efficacious than 10 μM H_2_S.

In the second set of experiments, LCS-1-SH adducts were prepared by pre-incubating low concentrations of H_2_S (10 μM or 50 μM) with 10 μM LCS-1, without or with 0.1 μM SOD1, for 30 min in closed containers to minimize H_2_S loss due to volatility. The containers were then opened for 1 h to allow volatilization of the unreacted H_2_S. The LCS-1-SH adduct without SOD1 was then incubated with SOD1 for another 30 min before adding H_2_S and SSP4. These samples were compared to controls preincubated for similar intervals but without H_2_S (Supplemental Figure S4B). There was no difference in polysulfide production between preincubating LCS-1 with SOD1 for 30 min prior to adding SSP4 and 300 μM H_2_S or adding SOD1 to LCS-1 immediately followed by SSP4 and 300 μM H_2_S. However, preincubation of LCS-1 with 10 μM H_2_S decreased polysulfide production by 35%, and preincubation of LCS-1 and SOD1 with 10 μM H_2_S decreased polysulfide production by 21%. With 50 μM H_2_S, these values were 21% and 19%, respectively. The increase in polysulfide production over time did not appear to be substantially different if LCS-1 was preincubated without or with SOD1 for any H_2_S treatment (red, green, and blue traces).

Collectively, these results suggest that LCS-1-SH adducts inhibit LCS-1 binding to SOD1, albeit less effectively than either LCS-1-GSH or LCS-1-Cys adducts. They also suggest that LCS-1 and SOD1 rapidly react with each other prior to augmenting H_2_S oxidation.

To confirm that there was little delay between addition of LCS-1 and SOD1 and enhancement of H_2_S oxidation, either 1 μM or 10 μM LCS-1 was incubated with 0.1 μM SOD1 0, 30, 60, 90, or 120 min before addition of H_2_S and SSP4 and fluorescence measured over an additional 130 min. As shown in Supplemental Figure S5A,B, the period of LCS-1 incubation with SOD1 did not affect either the onset or magnitude of polysulfide production. However, with 1 μM LCS-1, the delay before polysulfide production began to increase (approximately 70 min) was essentially twice that with 10 μM LCS-1 (~35 min). These results confirm that there is little, if any, delay between the addition of LCS-1 to SOD1 and the increased catalytic activity of the LCS-1-SOD1 complex.

### 3.8. SOD Inhibition by LCS-1 and ATN-244

Concentration-dependent inhibition of superoxide production by SOD1 using the nitro blue tetrazolium assay is shown in Supplemental Figure S6A. The approximate IC_50_ was 60 nM SOD. The effects of LCS-1 and the Cu-chelator ATN-244 on SOD activity are shown in Supplemental Figure S6B and the percent SOD1 inhibition in Supplemental Figure S6C. The addition of 10 μM ATN-244 nearly completely inhibited SOD1 activity, and the effects of ATN-244 were concentration-dependent. Conversely, all LCS-1 concentrations, from 0.1 μM to 100 μM, inhibited SOD1 by approximately 15%, and there was no apparent concentration-dependent effect. With the WST1 assay, 10 and 20 μM LCS-1 inhibited SOD1 by 22 and 35%, respectively, but these were not significantly different from each other (Supplemental Figure S6D). Collectively, it was not possible to inhibit more than 35% of SOD1 with LCS-1. This contrasts with an estimated 75% inhibition observed by Somwar et al. at 10 μM LCS-1 [2]. The reason for this large discrepancy is unknown, but the present experiments suggest that even with relatively high LCS-1 concentrations, SOD1 retains considerable dismutase activity.

### 3.9. Effects of LCS-1 on RSS in HEK293T Cells

**Figure 6:**
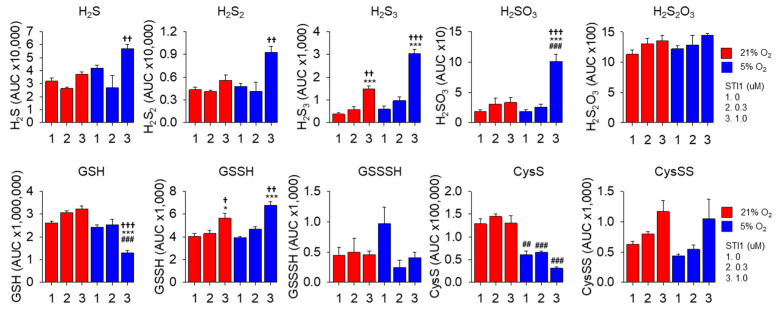
LC-MS/MS analysis of RSS in HEK293 cells after 48 h incubation in 21% (red bars) or 5% oxygen (blue bars) without or with 0.3 or 1 μM LCS-1. Mean + SE, n = 3 replicates; *, *p* < 0.05; ***, *p* < 0.001, 1 vs. 2 or 3 for either 25% or 5% oxygen; ##. *p* < 0.01; ###, *p* < 0.001 for 21% vs. respective 5% oxygen; †, *p* < 0.05; ††, *p* < 0.01; †††, *p* < 0.001 for 2 vs. 3.

As proof of principle, the effects of LCS-1 were examined in HEK293T cells. As shown in Figure 6, the effects of LCS-1 were more pronounced in 5% oxygen and with 1 μM LCS-1. Here, LCS-1 increased H_2_S, H_2_S_2_, H_2_S_3_, H_2_SO_3_, and GSSH and decreased GSH and Cys. The increases in inorganic polysulfides are generally consistent with LCS-1 oxidation of H_2_S in buffer, although they could also be the result of increased H_2_S due to decreased oxidation as observed with cells in 5% oxygen. Additional studies are underway to clarify these issues.

## 4. Discussion

Our experiments show that LCS-1 oxidizes H_2_S and that it acts synergistically with SOD1 to oxidize H_2_S to polysulfides with a preponderance of H_2_S_3-5_. These reactions have a slow onset but increase after 15–25 min, suggesting a chain reaction. H_2_S oxidation by the combination of LCS-1 and SOD1 consumes oxygen and produces hydrogen peroxide, but it does not appear to involve redox cycling of LCS-1. Nor does the hydrogen peroxide produced in these reactions appear to make a substantial contribution to polysulfide production. H_2_S oxidation is favored as the LCS-1:SOD1 ratio approaches or exceeds 10:1 or higher, and it is effective with as little as 0.01 μM SOD. LCS-1 forms monothiol adducts with H_2_S, GSH, and Cys, and these adducts inhibit LCS-1-SOD1 synergism. However, SOD1 was not completely inhibited by LCS-1. This suggests that in the observed reactions with H_2_S, SOD1 may have both oxidase and dismutase activities, but the relative proportions of each remain to be determined. In general, the LCS-1-SOD1 reactions observed in buffer were consistent with the effects of LCS-1 in cells, suggesting that some (if not many) of the effects of LCS-1 in biological systems may be mediated through thiol metabolism.

Quinones and naphthoquinones undergo consecutive one-electron reactions to oxidize H_2_S to polysulfides through redox cycling of the quinone with oxygen and H_2_S [14,22,23]. These reactions are also enhanced by SOD1. However, it is thought that in these reactions, SOD dismutation (and removal) of superoxide helps drive the otherwise unfavorable one-electron oxidation of the reduced hydroquinone by oxygen [25]. Since there was no evidence for LCS-1 redox cycling in the present studies, alternative catalytic mechanisms need to be considered.

### 4.1. H_2_S Oxidation by LCS-1

These experiments suggest that LCS-1 can oxidize H_2_S in reactions that consume H_2_S and molecular oxygen and produce polysulfides and hydrogen peroxide. There was no evidence that LCS-1 oxidation of H_2_S involved either one-electron production of LCS-1 radicals or a two-electron reduced quinone/hydroquinone-type molecule that could redox cycle with oxidized LCS-1. This could suggest that H_2_S oxidation by LCS-1 is distinct from redox cycling previously observed for quinones or naphthoquinones [14,22,23]. It is also possible that reduced LCS-1 was not detected due to rapid reoxidization. An S-adduct of LSC-1 formed through a Michael-type addition-elimination of Cl at the 4-position may also have redox activity. The structurally related herbicide chloridazon, which is a 4-amino (NH_2_) adduct of the LCS-1 scaffold (4-amino-5-chloro-3(2*H*)-pyridazinone), has useful redox properties [26], suggesting the potential for similar properties for the S-adduct of LCS-1.

### 4.2. Proposed Mechanism of LCS-1/SOD1 Synergism

The premise for LCS-1-SOD1 oxidation of H_2_S is based on previous observations that SOD1 can oxidize low molecular weight thiols such as Cys and, to a lesser extent, GSH. These reactions produce an oxidized dithiol and hydrogen peroxide Equation (1) but are independent of SOD1 dismutase activity [27].

2RSH + O_2_ + (SOD1) –> RSSR + H_2_O_2_ (1)

The reaction appears to be a two-step process. First, the low molecular weight thiol reduces the intramolecular Cys^57^-Cys^146^ disulfide bond in SOD1, which results in misfolded protein [28]. Misfolding opens the catalytic site, favors the loss of structural Zn^2+^, and, by increasing the oxidizing properties of Cu, changes SOD1 from a dismutase to an oxidase (Equation (2); [29,30]). In the second step, the misfolded SOD1 then catalytically oxidizes low molecular weight thiols and reduces molecular oxygen to H_2_O_2_. This reaction is especially efficient with Cys, where Cys readily reduces the intramolecular disulfide, and the resultant oxidized cystine is reduced back to Cys by GSH. The Cys keeps the intramolecular disulfide from reforming, i.e., a “cysteine-dependent redox short circuit” (Equation (3), [29]). The direct reaction of SOD1 with GSH is reportedly slow or non-existent [27,29], but its consumption through cystine reduction can deplete intracellular GSH and thereby exacerbate oxidative stress.

SOD1(Zn)(Cys^57^-Cys^146^) + 2Cys-S –> SOD1(Cys^57^, Cys^146^) + Cys-SS-Cys + Zn^2+^ (2)

Cys-SS-Cys + 2GSH –> 2Cys + GSSG (3)

Thiol oxidation appears to result, via an intermediate Equation (4), from a two-electron process whereby oxygen reoxidizes Cu^1+^ to Cu^2+^ and forms a sulfenic acid, the latter then reacting with another thiol to form the oxidized dithiol. Superoxide is not required in the following reactions (Equations (5) and (6); where R = Cys, [27]):

SOD1-Cu^2+^ + RSH <–> [SOD1-Cu^2+^-RS^−^ <–> SOD1-Cu^1+^-RS•] + H^+^ (4)

[SOD1-Cu^+1^-RS•] + O_2_ + H^+^ + H_2_S –> SOD1-Cu^2+^ + RSOH + H_2_O_2_ (5)

RSH + RSOH –> RSSR + H_2_O (6)

It is well known that LCS-1 binds to SOD1 [2] and increases intracellular ROS [3,4,6,7]. However, the mechanism of SOD1 inhibition by LCS-1 has not been resolved, i.e., there is no evidence that it directly binds to and inhibits redox-active Cu. The present studies suggest that LCS-1 forms an adduct with one (or both) of the intramolecular Cys^57^-Cys^146^ in SOD1, similar to Cys. By breaking this bond, SOD1 becomes an oxidase that enzymatically catalyzes the oxidation of H_2_S and reduces molecular oxygen as described for other thiols.

Collectively, the present experiments support the above hypothesis. LCS-1 and SOD1 act synergistically to oxidize H_2_S to polysulfides. This reaction consumes oxygen and produces ROS, but neither superoxide nor hydrogen peroxide appears to substantially contribute to H_2_S oxidation. This synergism is inhibited by preincubation of LCS-1 with GSH or Cys, presumably because the LCS-1-GSH or LCS-1-Cys adduct can no longer reduce the Cys^57^-Cys^146^ disulfide in SOD1. Furthermore, the sulfenic acid produced in this reaction can also react with polyhydrosulfides to produce longer polyhydrosulfides (Equation (7)).

H_2_S_n_ + HSOH –> H_2_S_(n+1)_ + H_2_O (7)

### 4.3. Chemical Reactivity of Products from H_2_S Oxidation

The hallmark of LCS-1 activity in cell toxicity is increased ROS production and depletion of GSH [2,3,4,7,8,29]. This is thought to occur somewhat paradoxically via an increase in hydrogen peroxide, although it could be due to GSH depletion. The primary reactive products of H_2_S oxidation by LCS-1/SOD1 are inorganic per- and polysulfides (H_2_S_2_ and H_2_S_3-6_), sulfite, and polysulfenic acids. How these act and interact in the context of biological systems is yet to be fully resolved, especially regarding their role(s) as cellular oxidants or reductants and in the context of homeostatic signaling molecules or cytotoxic compounds [31]. In arguably the most simplistic form, organic per- and polysulfides (where R is generally GSH or Cys and R’ may be GSH, Cys, or protein-Cys) are often considered in equilibrium with H_2_S or its anion HS^−^ (Equation (8)).

H_2_S(HS^−^) + RSSR’ <–> RSSH(RSS^−^) + R’SH (8)

The biological outcome of this reaction will depend on what products are favored. Inorganic and organic hydroper- and hydropolysulfides may be either nucleophiles or electrophiles [32,33]. It has been argued that the reaction shown in eq. 8 is favored far to the left and that RSSH(RSS^−^), if present, functions as potent electrophiles. This is based on thermodynamic grounds and practical considerations, i.e., protein integrity would be lost if their disulfide bonds were readily oxidized by H_2_S [33]. On the other hand, while acknowledging that the reaction in Equation (11) is favored to go to the left, it has also been posited that “unlike RSH, RSSH can be either reductants/nucleophiles or oxidants/electrophiles depending on the protonated state” [32].

**Figure 7:**
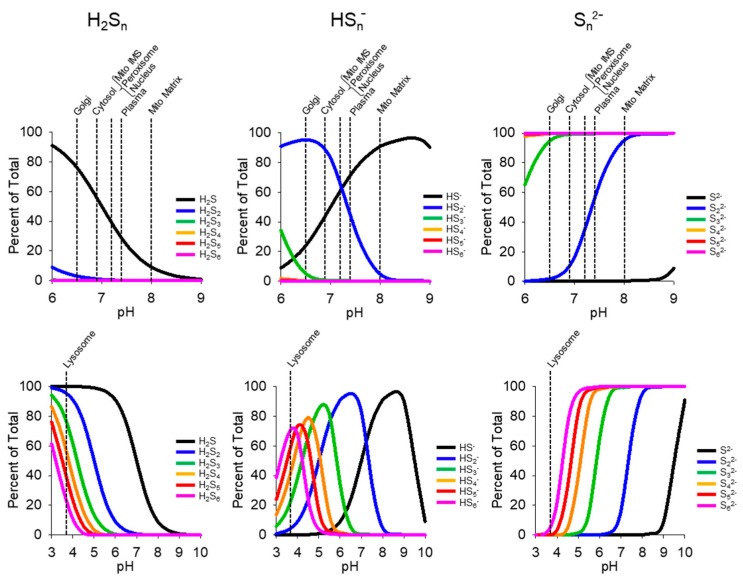
Effect of pH on the percent distribution of protonated (H_2_S_n_), anionic (HS_n-_), and dianionic (S_n_^2−^) sulfur species as a function of the number of catenated sulfur atoms (n). Dashed lines indicate the approximate pH of various cellular compartments and plasma.

The pKa_1_ and pKa_2_ of inorganic per- and polysulfides decrease as the number of sulfur atoms increases Table S1 Supplemental information [34]. This progressively increases the degree of ionization (Figure 7). At pH 7, half of S_1_ is fully protonated (H_2_S) and half is the hydrosulfide anion (HS^−^), whereas with S_2_, less than 1% is protonated, nearly 83% is the hydrosulfide anion, and over 16% is the dianion. The pKa for small organic thiols also decreases below 6.0 upon persulfidation, e.g., 5.45 for GSSH and 5.2 for CysSSH, reportedly increasing their nucleophilicity and reactivity [35,36].

While the pH of intracellular organelles will affect the degree of ionization, it is evident that, except for the acidic lysosomes, essentially all intracellular S_3-6_ will be dianions and 20% of small organic thiols will be anions. This suggests an increased propensity for nucleophilic interactions, which would help maintain a reduced intracellular environment, but at the risk of destabilizing disulfide bridges. However, based on our LC-MS/MS analysis of the products of H_2_S oxidation by LCS-1 and LCS-1-SOD1, it also appears that the amount of polysulfides produced in these reactions decreases exponentially as the number of sulfur atoms increases. pH-specific intercompartmental equilibria of polysulfides, which favor more permeable, fully protonated species, will further complicate the issue. How these factors interact and affect the sulfur biome is a challenging and difficult task.

### 4.4. Biological Significance of H_2_S Oxidation by LCS-1

Here, we demonstrate that LCS-1 impacts low molecular weight RSS in HEK293T cells, and we show that these effects are consistent with H_2_S oxidation by LCS-1 and LCS-1/SOD1 in buffer. These off-target actions are likely to affect cellular sulfur metabolism and signaling, and they suggest alternative mechanisms need to be considered in evaluating the therapeutic efficacy of LCS-1. While the present work was designed as proof of principle, it clearly demonstrates the need for a reappraisal of the biological actions of LCS-1 in other cells and contexts.

## Data Availability

The data presented in this study are available on request from the corresponding author.

## Supplementary Materials

The following supporting information can be downloaded at: https://www.mdpi.com/article/10.3390/antiox13080991/s1.
