# Supplementary material for: The SOD1 Inhibitor, LCS-1, Oxidizes H2S to Reactive Sulfur Species, Directly and Indirectly, through Conversion of SOD1 to an Oxidase
Source: Antioxidants (Basel). 2024 Aug 15;13(8):991. doi: 10.3390/antiox13080991 (PMC11351665; doi:10.3390/antiox13080991)
Supplement: Supplementary file 1 [file antioxidants-13-00991-s001.zip › Olson et al Supplemental Materials.pdf]

**The SOD1 Inhibitor, LCS-1, Oxidizes H2S to Reactive Sulfur Species, Directly and Indirectly, Through Conversion of SOD1 to an Oxidase**

Kenneth R. Olson [1,2,*], Tsuyoshi Takata [1], Kasey J. Clear [3], Yan Gao [1], Zhilin Ma [1,2], Ella Pfaff [1,2], Karthik Mouli [4], Thomas A Kent [4], Prentiss Jones, Jr. [5], Jon Fukuto [6], Gang Wu [7] and Karl D. Straub [8,9]

[1] Department of Physiology, Indiana University School of Medicine South Bend, South Bend, IN 46617, USA
[2] Department of Biological Sciences, University of Notre Dame, Notre Dame, IN 46556, USA
[3] Department of Chemistry and Biochemistry, Indiana University South Bend, South Bend, IN 46615, USA
[4] Institute of Biosciences and Technology, Texas A&M Health Sciences Center-Houston Campus, Houston, TX 77030, USA
[5] Toxicology Department, Western Michigan University Homer Stryker M.D. School of Medicine, Kalamazoo, MI 49007, USA
[6] Department of Chemistry, Sonoma State University, Rohnert Park, CA 94928, USA
[7] Department of Internal Medicine, McGovern Medical School, University of Texas, Houston, TX 77030, USA
[8] Central Arkansas Veteran's Healthcare System, Little Rock, AR 72205, USA
[9] Departments of Medicine and Biochemistry, University of Arkansas for Medical Sciences, Little Rock, AR 72202, USA
* Correspondence: kolson.1@nd.edu; Tel.: +574-631-7560; Fax: +574-631-7821

**Running Head: Sulfur Metabolism by LCS-1**

***Address correspondence to:**
Kenneth R. Olson, Ph.D.
Indiana University School of Medicine -South Bend
Raclin Carmichael Hall
1234 Notre Dame Avenue
South Bend, IN 46617
**Phone:** (574) 631-7560
**Fax:** (574) 631-7821
**e-mail:** olson.1@nd.edu

**Inorganic sulfur speciation**

The percent ionization of inorganic RSS ($H_2S_{1-6}$) was calculated at pH increments from 3 to 10 using Henderson Hasselbach equations (eqs. 1, 2) and the dissociation constants in **Table 1**. In this case with $S_1$ as an example,

$$pH - pKa_1 = Log_{10}[HS^-]/[H_2S] \quad \textit{(eq. 1)},$$

$$pH - pKa_2 = Log_{10}[S^{2-}]/[HS^-] \quad \textit{(eq. 2)}.$$

At any specified pH, if X equals antilog$_{10}$(pH - pKa$_1$) and Y equals antilog$_{10}$(pH - pKa$_2$) then,

$$X = [HS^-]/[H_2S] \quad \textit{(eq. 3)},$$

and,

$$Y = [S^{2-}]/[HS^-] \quad \textit{(eq. 4)},$$

Solving eqs. 3 and 4 for $H_2S$ and $S^{2-}$,

$$[H_2S] = [HS^-]/X \quad \textit{(eq. 5)},$$

$$[S^{2-}] = [HS^-]*Y \quad \textit{(eq. 6)}.$$

Letting the total sulfur species,

$$H_2S + HS^- + S^{2-} = 1 \quad \textit{(eq. 7)},$$

and substituting for $H_2S$ and $S^{2-}$ (eqs. 5 and 6),

$$HS^-/X + HS^- + HS^-*Y = 1 \quad \textit{(eq. 8)},$$

$HS^-$ is then,

$$HS^- = 1/(1/X + 1 + Y) \quad \textit{(eq. 9)},$$

and $HS^-$ can be calculated at any pH using X and Y for that pH. Knowing $HS^-$, $H_2S$ and $S_2^-$ can then be calculated at that pH by rearranging eqs. 3 and 4 and the percent of each species can be calculated from the total $H_2S + HS^- + S_2^-$.

**Table 1.** $pKa_1$ and $pKa_2$ of catenated sulfur species[a].

| Species | $pKa_1$ | $pKa_2$ |
|---|---|---|
| $S_1$ | 7.0 | >14 |
| $S_2$ | 5.0 | 9.7 |
| $S_3$ | 4.2 | 7.5 |
| $S_4$ | 3.8 | 6.3 |
| $S_5$ | 3.5 | 5.7 |
| $S_6$ | 3.2 | 5.2 |

a, From [1]

## References

1. Kamyshny, A., Jr.; Goifman, A.; Rizkov, D.; Lev, O., Formation of carbonyl sulfide by the reaction of carbon monoxide and inorganic polysulfides. *Environ. Sci. Technol* **2003,** 37, (9), 1865-1872.

**Supplemental Figure Captions**

**Supplemental Figure S1.** Absorption spectra of LCS-1 reactions with $H_2S$ and effects of SOD, cysteine (Cys) and hydrogen peroxide ($H_2O_2$), reactant concentrations are shown above each panel. (**A-D**) Spectra of individual reactants, (**E- K**) Spectra with DMSO spectrum subtracted. Scale to right indicates elapsed time, $H_2S$ and Cys were added after 10 s, arrows mark absorption peaks and wavelength (nm). $H_2S$ did not affect the LCS-1 spectrum.

**Supplemental Figure S2.** LC-MS/MS analysis of products after 30 min incubation of 300 µM $H_2S$ with 10 µM LCS-1 and 0.1 µM SOD1 without or with 1 µM catalase (Cat). Cat slightly decreased $H_2S_{2\text{-}4}$ and $H_2SO_3$, increased $H_2S_2O_3$, whereas $H_2S$, $H_2S_{5,6}$ and $HS_{4,5}OH$ were unaffected. Mean +SE, n=3 replicates; *, *p<0.05*; ***, *p<0.001* versus without Cat.

**Supplemental Figure S3.** Effects of LCS-1-thiol adducts on $H_2S$ oxidation by SOD1. (**A,B**) Effects of 30 min pre-incubation with GSH (**A**) or Cys (**B**) on polysulfide production (SSP4 fluorescence) from oxidation of 300 µM $H_2S$ by 10 µM LCS-1 and 0.1 µM SOD1. Pre-incubation with equimolar (10 µM) GSH increased polysulfides compared to LCS-1-SOD1, whereas 50 µM GSH almost completely inhibited it, polysulfide production was inhibited by both 10 µM and 50 µM Cys. Little polysulfides were produced by 30 min preincubation with GSH or Cys in the absence of either SOD1 or LCS-1. Mean +SE, n=4 wells; ***, *p<0.001* versus $H_2S$-LCS-1-SOD (no adduct); #, *p<0.05*, ###, *P<0.001*, 10 µM versus respective 50 µM GSH or Cys.

**Supplemental Figure S4.** Effects of LCS-1-SH adducts on $H_2S$ oxidation by SOD-1. (**A**) Preincubation of 10 μM LCS-1 or 10 μM LCS-1 plus 0.1 μM SOD1 for 10 min with either 10 μM or 50 μM $H_2S$ followed by 300 μM $H_2S$ and SSP4 compared to LCS-1 plus SOD pre-incubated for 0 or 10 min without $H_2S$. (**B**) $H_2S$ (10 μM or 50 μM) was incubated in covered containers with 10 μM LCS-1 without or with 0.1 μM SOD for 30 min, the covers were then removed for 1 h to let un-reacted $H_2S$ dissipate and 0.1 μM SOD1 added, the LCS-1-SH adduct without SOD1 was then incubated with SOD1 for another 30 min before adding $H_2S$ and SSP4. These samples were compared to controls preincubated for similar intervals but without $H_2S$. All $H_2S$ adducts decreased polysulfide production. Bar graphs summarize results at 140 min (**A**) or 120 min (**B**), line graphs show responses over time. Mean +SE, n=4 wells per treatment; ***, *p<0.001* versus respective LCS-1 plus SOD1 without $H_2S$; ##, *p<0.01*, ###, *p<0.001*, 10 μM $H_2S$ versus 50 μM $H_2S$ preincubation.

**Supplemental Figure S5.** Effects of duration of incubation of either 1 μM (**A**) or 10 μM (**B**) LCS-1 with 0.1 μM SOD1, prior to addition of 300 μM $H_2S$, on polysulfide production (SSP4 fluorescence). LCS-1 was incubated with SOD1 0, 30, 60, 90, or 120 min before addition of $H_2S$ and SSP4 and fluorescence measured over an additional 130 min. The onset of polysulfide production was slower with 1 μM LCS-1 than with 10 μM LCS-1 but duration of LCS-1 incubation with SOD1 did not significantly affect polysulfide production suggesting that LCS-1 rapidly reacts with SOD1. Mean +SE, n=4 wells per treatment.

**Supplemental Fig. S6.** (**A-C**) Effects of LCS-1 and ATN-244 on SOD1 inhibition using the nitro blue tetrazolium assay. (**A**) Standard curve of SOD1 inhibition, approximate $IC_{50}$ was 0.06

μM SOD and nearly complete inhibition was obtained with 1 μM SOD. (**B-C**) ATN-244 concentration-dependently inhibited SOD1 with nearly complete inhibition at 10 μM. Conversely, LCS-1 only inhibited SOD1 by approximately 15% and there was little difference between 0.1 and 100 μM LCS-1. (**D**) LCS-1 inhibition of SOD1 using the WST1 assay. Ten and 20 μM LCS-1 inhibited SOD1 by 22 and 35%, respectively but they were not significantly different from each other. Mean +SE, n=4 (nitro blue assay) or n=3 (WST1 assay) wells; *, $p<0.05$; **, $p<0.01$; ***, $p<0.001$ versus SOD alone.
