# Supplementary figures and images for: The SOD1 Inhibitor, LCS-1, Oxidizes H2S to Reactive Sulfur Species, Directly and Indirectly, through Conversion of SOD1 to an Oxidase
Source: Antioxidants (Basel). 2024 Aug 15;13(8):991. doi: 10.3390/antiox13080991 (PMC11351665; doi:10.3390/antiox13080991)

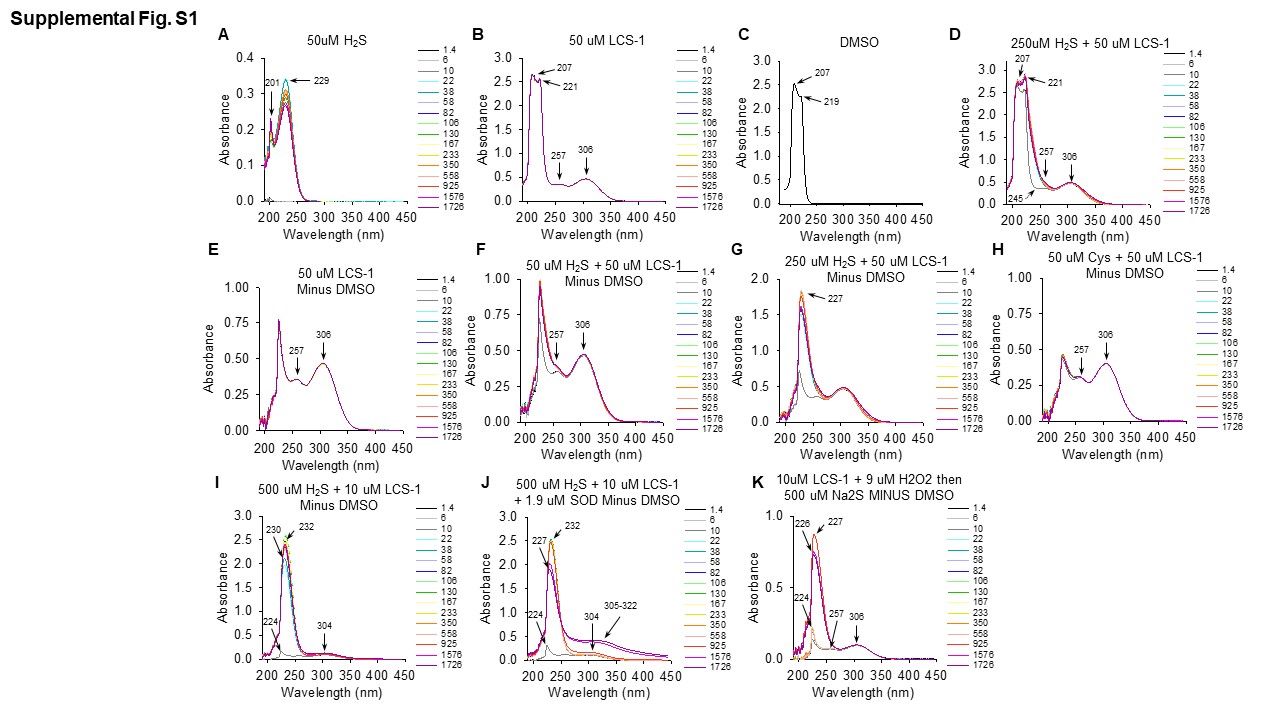

Supplement: Supplementary file 1 [file antioxidants-13-00991-s001.zip › Slide1.JPG]

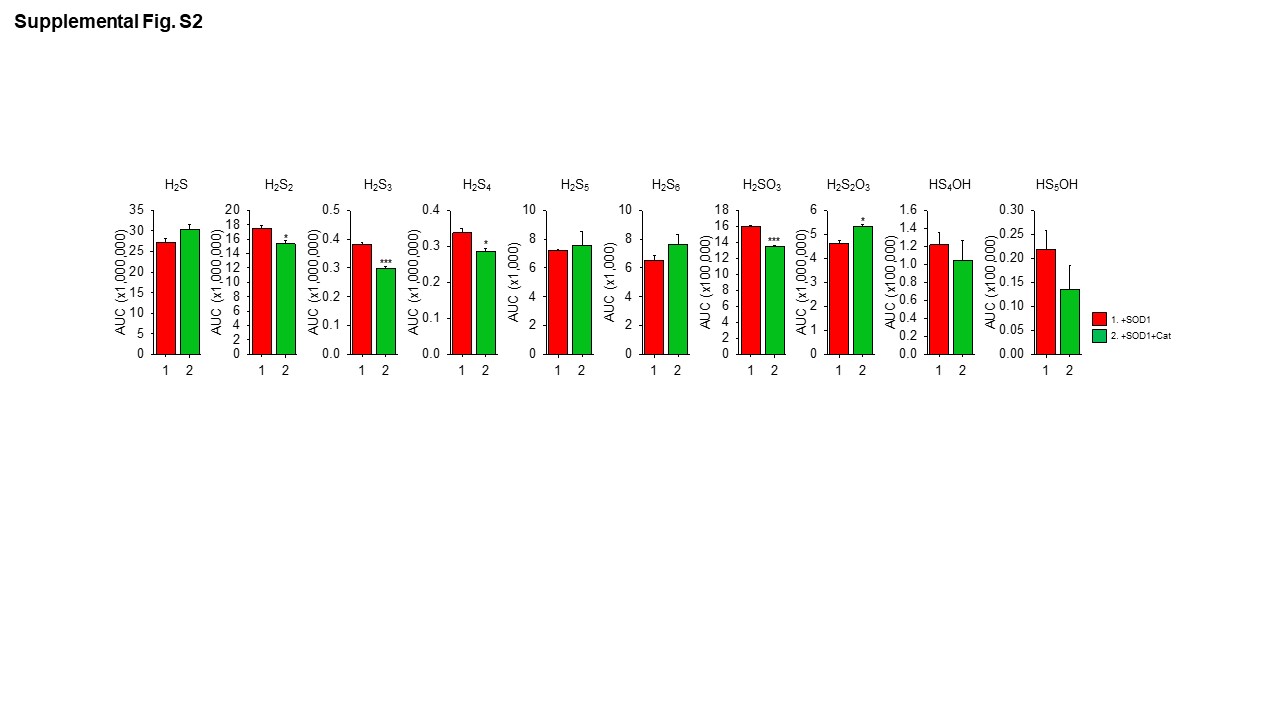

Supplement: Supplementary file 1 [file antioxidants-13-00991-s001.zip › Slide2.JPG]

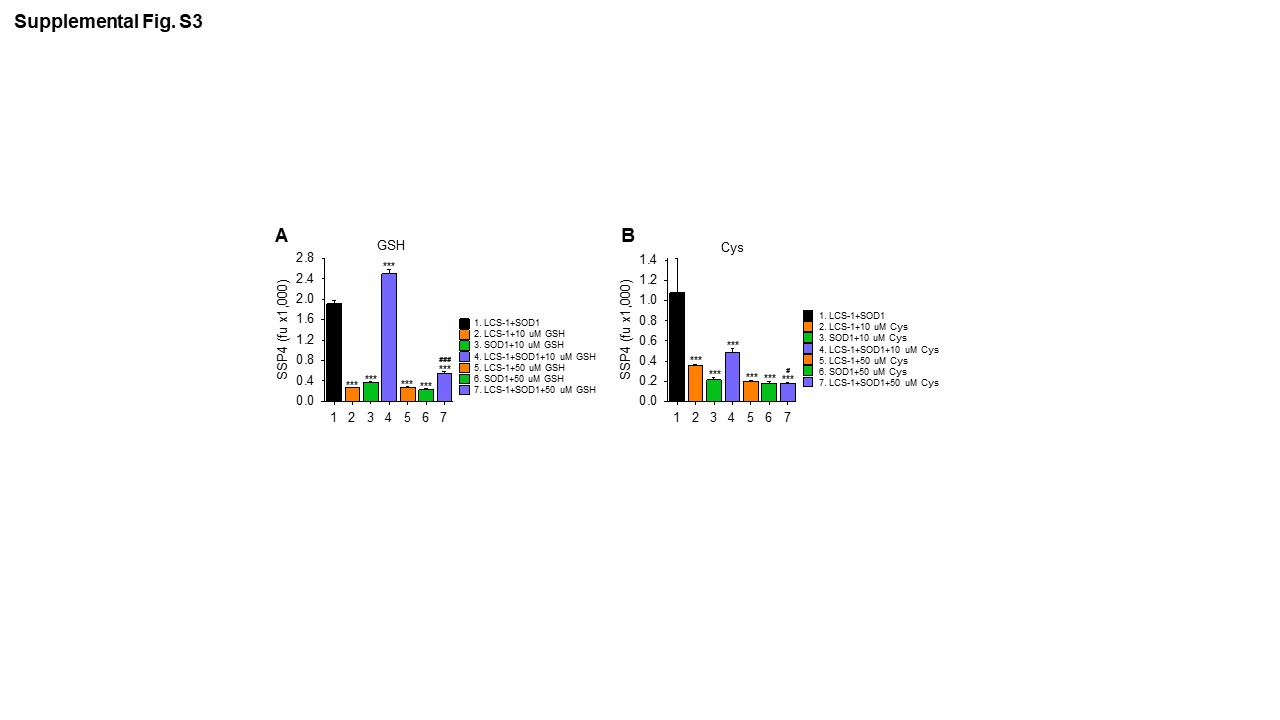

Supplement: Supplementary file 1 [file antioxidants-13-00991-s001.zip › Slide3.JPG]

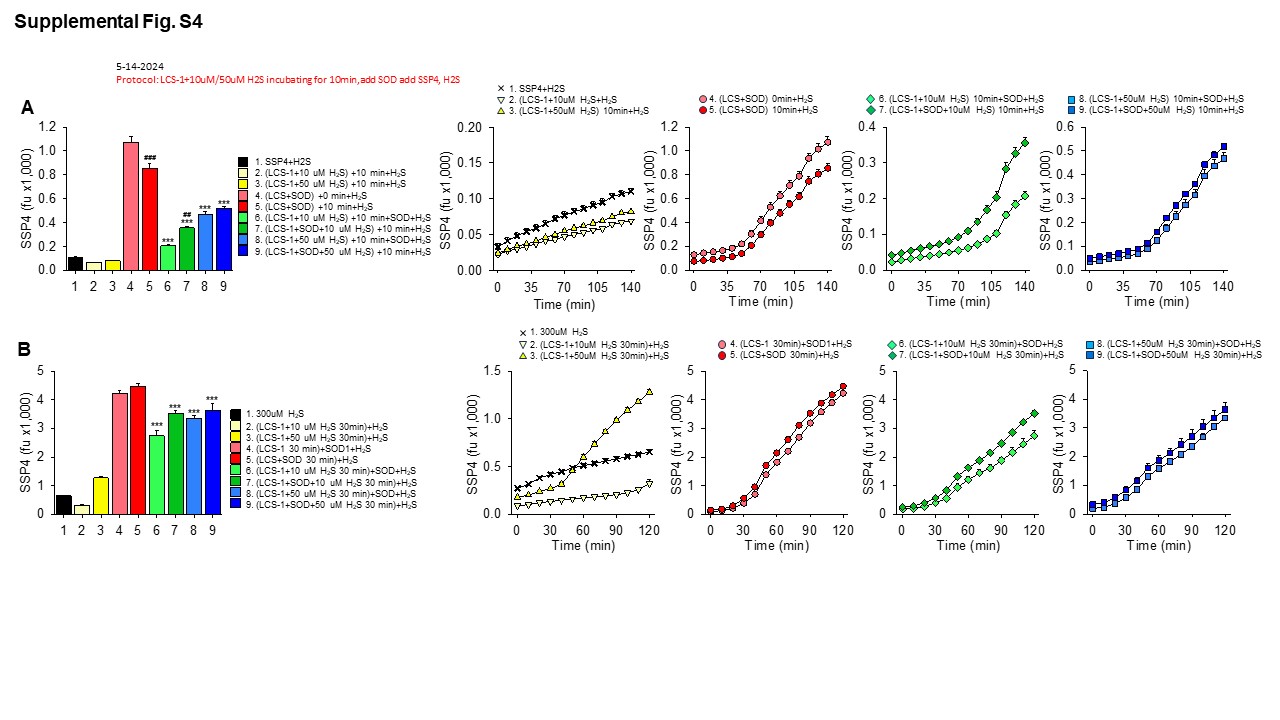

Supplement: Supplementary file 1 [file antioxidants-13-00991-s001.zip › Slide4.JPG]

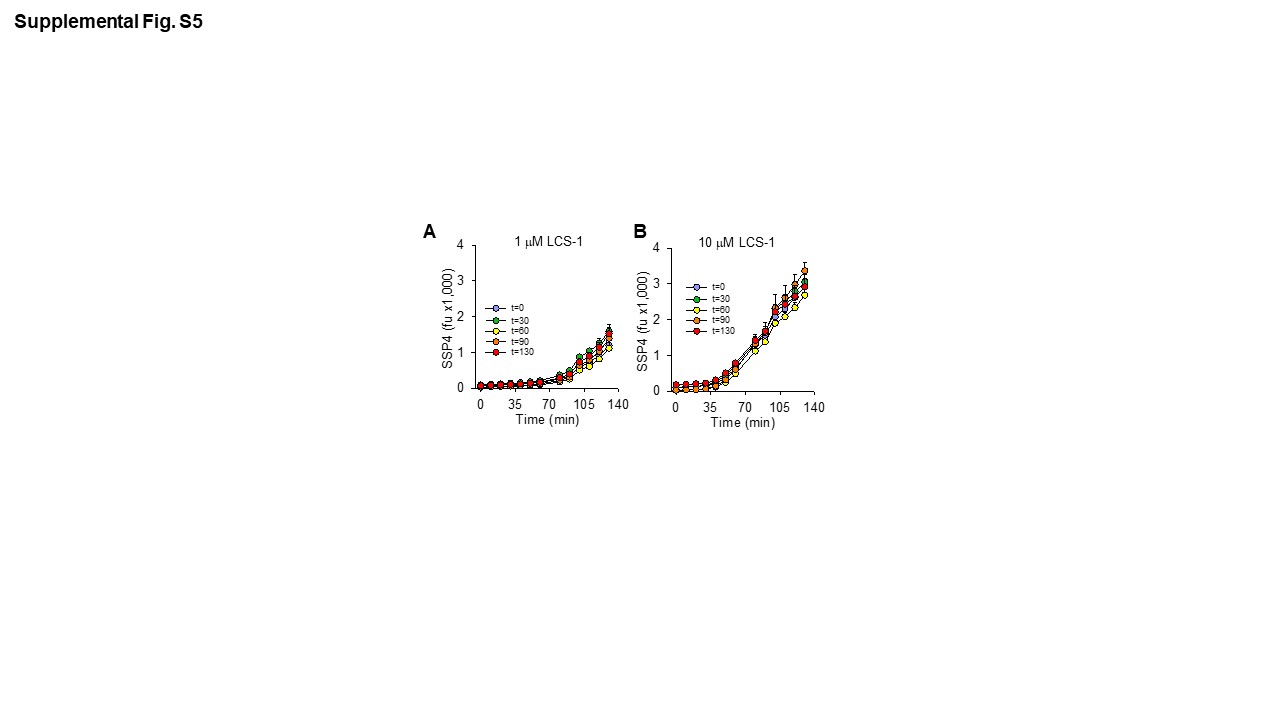

Supplement: Supplementary file 1 [file antioxidants-13-00991-s001.zip › Slide5.JPG]

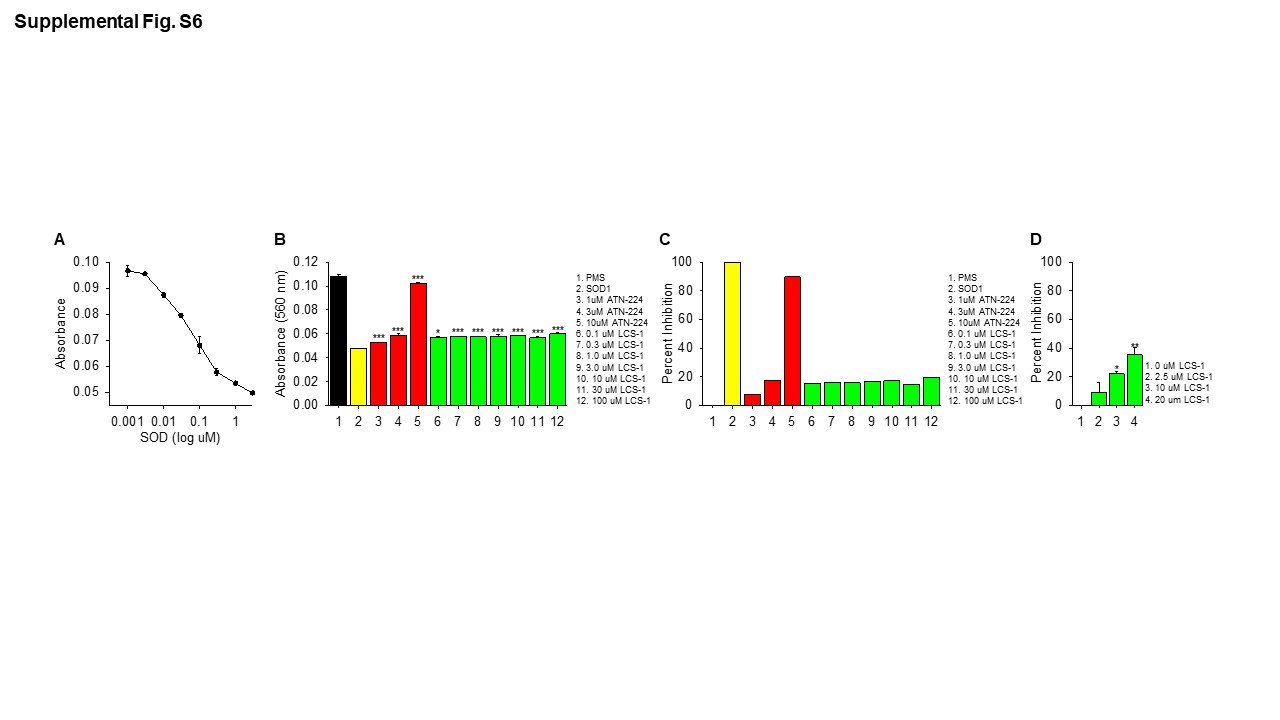

Supplement: Supplementary file 1 [file antioxidants-13-00991-s001.zip › Slide6.JPG]
